# Cold Exposure Drives Weight Gain and Adiposity following Chronic Suppression of Brown Adipose Tissue

**DOI:** 10.3390/ijms23031869

**Published:** 2022-02-07

**Authors:** Peter Aldiss, Jo E. Lewis, Irene Lupini, Ian Bloor, Ramyar Chavoshinejad, David J. Boocock, Amanda K. Miles, Francis J. P. Ebling, Helen Budge, Michael E. Symonds

**Affiliations:** 1 Academic Unit of Population and Lifespan Sciences, Centre for Perinatal Research, School of Medicine, University of Nottingham, Nottingham NG7 2UH, UK; ian.bloor@nottingham.ac.uk (I.B.); ramyar.chavoshinejad@nottingham.ac.uk (R.C.); helen.budge@nottingham.ac.uk (H.B.); 2 Section for Nutrient and Metabolite Sensing, The Novo Nordisk Foundation Center for Basic Metabolic Research, University of Copenhagen, 2200 Copenhagen, Denmark; 3 Queen’s Medical Centre, School of Life Sciences, University of Nottingham, Nottingham NG11 8N, UK; jl2033@medschl.cam.ac.uk (J.E.L.); fran.ebling@nottingham.ac.uk (F.J.P.E.); 4 School of Biosciences and Veterinary Medicine, University of Camerino, 62032 Camerino, Italy; Irene.lupini@studenti.unicam.it; 5 John van Geest Cancer Research Centre, Nottingham Trent University, Nottingham NG11 8N, UK; david.boocock@ntu.ac.uk (D.J.B.); amanda.miles@ntu.ac.uk (A.K.M.); 6 Nottingham Digestive Disease Centre, Biomedical Research Center, School of Medicine, University of Nottingham, Nottingham NG11 8N, UK

**Keywords:** brown adipose tissue, thermoneutrality, healthy expansion of adipose tissue, proteomics

## Abstract

Therapeutic activation of thermogenic brown adipose tissue (BAT) may be feasible to prevent, or treat, cardiometabolic disease. However, rodents are commonly housed below thermoneutrality (~20 °C) which can modulate their metabolism and physiology including the hyperactivation of brown (BAT) and beige white adipose tissue. We housed animals at thermoneutrality from weaning to chronically supress BAT, mimic human physiology and explore the efficacy of chronic, mild cold exposure (20 °C) and β3-adrenoreceptor agonism (YM-178) under these conditions. Using metabolic phenotyping and exploratory proteomics we show that transfer from 28 °C to 20 °C drives weight gain and a 125% increase in subcutaneous fat mass, an effect not seen with YM-178 administration, thus suggesting a direct effect of a cool ambient temperature in promoting weight gain and further adiposity in obese rats. Following chronic suppression of BAT, uncoupling protein 1 mRNA was undetectable in the subcutaneous inguinal white adipose tissue (IWAT) in all groups. Using exploratory adipose tissue proteomics, we reveal novel gene ontology terms associated with cold-induced weight gain in BAT and IWAT whilst Reactome pathway analysis highlights the regulation of mitotic (i.e., G2/M transition) and metabolism of amino acids and derivatives pathways. Conversely, YM-178 had minimal metabolic-related effects but modified pathways involved in proteolysis (i.e., eukaryotic translation initiation) and RNA surveillance across both tissues. Taken together these findings are indicative of a novel mechanism whereby animals increase body weight and fat mass following chronic suppression of adaptive thermogenesis from weaning. In addition, treatment with a B3-adrenoreceptor agonist did not improve metabolic health in obese animals raised at thermoneutrality.

## 1. Introduction

Therapeutic activation of thermogenic brown adipose tissue (BAT) may be feasible to prevent, or treat, cardiometabolic disease [1]. In rodent models of obesity, the activation of BAT and uncoupling protein (UCP1)-positive beige adipocytes in white adipose tissue (WAT) by cold exposure and sympathomimetics (i.e., β3-agonists) can attenuate or reverse obesity, diabetes and atherosclerosis, thus improving metabolic health [1]. A major factor influencing these outcomes is that animals are typically housed at temperatures well below their thermoneutral zone (which for a rodent is c. 28 °C) [2]. Under these conditions, not only is BAT hyperactive, but UCP1+ beige adipocytes are readily seen in the inguinal WAT (IWAT) depot [3].

The ‘cold-stressed’ animal has been widely studied but much less is known about the underlying adaptations in adipose tissue of animals maintained at thermoneutrality. Usually, temperatures as low as 4 °C, which represent an ‘extreme cold’, are used to activate BAT, with the induction of UCP1 and subsequent thermogenic response primarily seen in subcutaneous IWAT and other ‘beige’ depots [3]. Conversely, when animals are housed at thermoneutrality and then exposed to 20 °C, the induction of UCP1 is primarily seen in BAT. These differences suggest there are two steps to the ‘browning’ process and emphasise the need to study rodent metabolism under thermoneutral conditions. Importantly, it was recently demonstrated that BAT from obese mice, housed chronically at thermoneutrality closely resembles human BAT [4]. This model of ‘physiologically humanised BAT’ is now thought to represent the best choice for studying the physiology of this key metabolic tissue and it has recently been shown that intermittent cold exposure of thermoneutrally housed animals exacerbates diet-induced obesity [5]. Here, we extend this recent work by raising animals at thermoneutrality, on an obesogenic diet from weaning. Beginning this early is a particularly important consideration given the early developmental steps which would be occurring in early life [6]. Furthermore, we use rats as classically, their physiology to external stressors such as diet and the environment is closer, than mice, to humans [7]. Using this model we have demonstrated UCP1 mRNA is absent in subcutaneous IWAT (the classical ‘beige’ depot) and not induced with exercise training [8], a common response at standard housing temperatures and one which is typically not seen in humans [9]. We hypothesised that activation of BAT by mild cold and YM-178, a highly selective β3-agonist that was recently been shown to activate BAT in lean and obese humans, and drive improvements in lipid metabolism, would be negligible under these conditions [10,11]. Moreover, we hypothesised that perivascular BAT (PVAT) which surrounds the thoracic aorta and plays a crucial role in vascular function, lipid metabolism and thermoregulation would be more responsive, as was previously shown to be the case with exercise training [8,12,13]. Finally, we sought to determine how the AT proteome responds following chronic suppression of BAT to better understand the molecular response to potentially thermogenic stimuli at thermoneutrality.

## 2. Results

Therapeutic activation of BAT by cold exposure drives weight loss and improves metabolic health. However, basal BAT activity is an undoubtedly an important consideration in the interpretation and translation of this field to humans. Animals housed at standard housing temperatures (i.e., 20–22 °C) have chronically active BAT, which exhibits supra-physiologically activity when animals are cold-exposed (i.e., to 4 °C).

**Figure 1:**
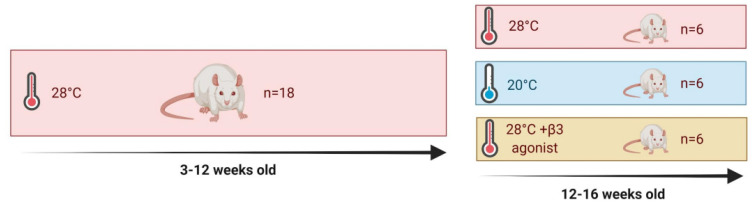
Experimental outline for study.

**Figure 2:**
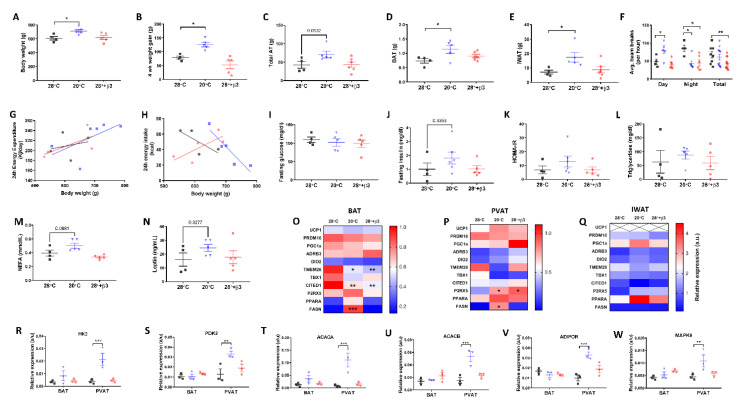
Cold exposure (20 °C) but not YM-178 (28 °C + β3) drove weight gain and deposition of BAT and inguinal white adipose tissue (IWAT) with no effect on serum metabolites or thermogenic markers. (**A**) Final body weight, (**B**) 4-week intervention weight gain, (**C**) total fat mass, (**D**) BAT mass, (**E**) IWAT mass, (**F**) 24 h ambulatory activity, (**G**) 24 h energy expenditure, (**H**) 24 h energy intake, (**I**–**N**) serum hormones and metabolites. (**O**–**Q**) Markers of brown and beige adipose tissue in BAT, PVAT and IWAT, (**R**–**W**) select metabolic genes in BAT and PVAT. Data expressed as mean ± SEM, n = 4–5 per group. For comparison, data was analysed by either one (**A**–**E**,**H**–**Q**), two-way ANOVA (**F**,**R**–**W**) or ANCOVA (**G**,**H**) with Sidak post hoc tests. Significance denoted as * *p* < 0.05; ** *p* < 0.01 or *** *p* < 0.001.

Modelling human physiology to ‘humanise’ BAT we raised animals at thermoneutrality (i.e., 28 °C) on high-fat diet from weaning to 12 weeks old. Four weeks of subsequent exposure to mild cold (i.e., 20 °C) promoted weight gain and increased BAT and subcutaneous IWAT mass, an effect not seen with a clinically relevant dose of the highly selective β3-agonist YM-178 (Figure 1 and Figure 2A–E) [14]. There was no detectable change in total fat mass (i.e., the total of all dissected depots), gonadal (GWAT), mesenteric (MWAT), retroperitoneal (RPWAT) or paracardial (PCAT) fat depots, or in liver or heart mass (Figure 2C and Figure S1B–G) suggesting cold does not drive whole-body changes in adiposity, or lean mass. There was however a significant increase in kidney size (Figure S1H). As there was no difference in 24 h energy expenditure, or intake, (Figure S1I,J) we incorporated these parameters, alongside body mass, into an ANCOVA. Whilst there was no evidence that changes in body mass were associated with altered energy expenditure (Figure 2G) in cold exposed rats there was a significant, unexpected relationship between body mass and energy intake in this group (r^2^ = 0.852, *p* = 0.025, Figure 2H) which was not seen in animals treated with YM-178. (Figure 2A–H). Increased weight gain and adiposity in cold-exposed animals was not associated with impaired metabolic parameters (i.e., serum glucose, triglycerides and NEFA) or hormones (i.e., insulin and leptin) (Figure 2I–N) suggesting weight gain was not pathological whilst there was no difference in corticosterone (Figure S1K) between groups suggesting no impact of cold exposure, or YM-178, on chronic stress.

Neither cold exposure, nor YM-178 were effective at inducing thermogenic genes (i.e., UCP1) in BAT or PVAT (Figure 2O–P) suggesting that chronic suppression of BAT from weaning reduces the ability of these thermogenic tissues to respond to a cold stimulus. Expression of the putative BAT and beige markers CITED1, and TMEM26, were reduced in BAT of cold exposed, and YM-178 treated rats, whilst the beige marker P2RX5 was upregulated in PVAT. Using targeted arrays to screen for primary genes involved in adipose tissue metabolism we saw an increase in the expression of FASN mRNA in both BAT and PVAT suggesting increased lipogenesis in both depots (Figure 2O,P). There was also an increase in genes involved in glycolysis (i.e., HK2 and PDK), fatty acid oxidation (i.e., ACACA and ACACB) and insulin resistance (i.e., AdipoR1 and MAPK9) in PVAT only following cold exposure (Figure 2R–W), demonstrating that this tissue is more responsive to cold than classic BAT when animals are raised at thermoneutrality. In contrast, in IWAT, UCP1 mRNA (Figure 2Q) was absent in all rats and, despite a c.125% increase in IWAT mass of cold exposed rats, there was no change in the expression of other genes associated with thermogenesis (i.e., ADRβ3), beige adipocytes (i.e., TMEM26) or lipogenesis (i.e., FASN) with this tissue being relatively unresponsive to all challenges.

**Figure 3:**
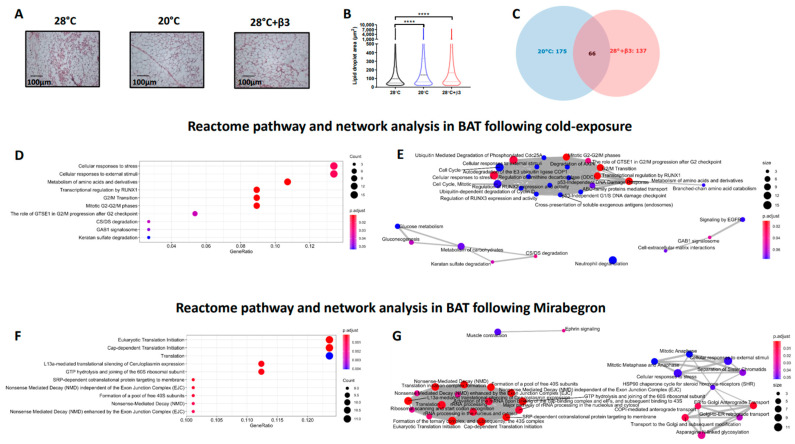
Histological and proteomics analysis of BAT following cold exposure (20 °C) and YM-178 treatment (28 °C + β3). (**A**,**B**) Histological analysis of BAT and lipid droplet area. (**C**) Venn diagram of differentially regulated proteins. Reactome pathway analysis detailing enriched pathways and interrelated networks in cold exposed (20 °C, **D**,**E**) and YM-178 treated animals (28 °C + β3, **F**,**G**). Data expressed as mean ± SEM, n = 4–5 per group. Adipocyte/lipid droplet area quantified using Adiposoft (28 °C, n = 8949; 20 °C, 15512 and 28 °C + β3, 12446). For comparison, data was analysed by one-way ANOVA (**B**) or using the ReactomePA package (**D**–**G**). Significance denoted as **** *p* < 0.0001.

**Figure 4:**
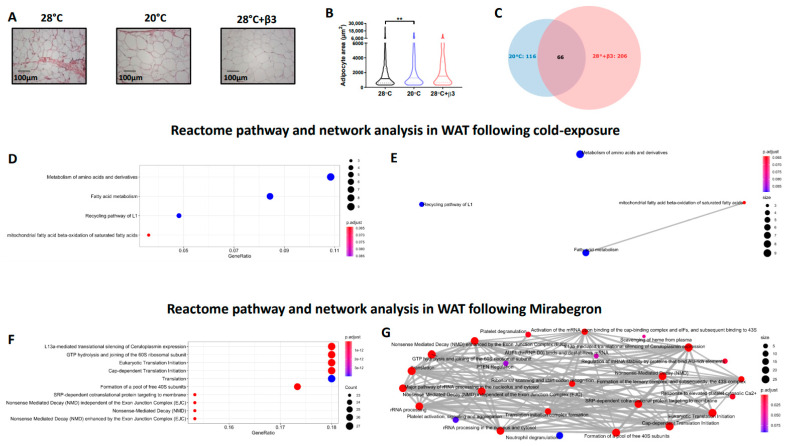
Histological and proteomics analysis of WAT following cold exposure (20 °C) and YM-178 treatment (28 °C + β3). (**A**,**B**) Histological analysis of BAT and lipid droplet area. (**C**) Venn diagram of differentially regulated proteins. Reactome pathway analysis detailing enriched pathways and interrelated networks in cold-exposed (20 °C, **D**,**E**) and YM-178 treated animals (28 °C + β3, **F**,**G**). Data expressed as mean ± SEM, n = 4–5 per group. Adipocyte/lipid droplet area quantified using Adiposoft (28 °C, n = 750; 20 °C, 553 and 28 °C + β3, 527). For comparison, data was analysed by one way ANOVA (**B**) or using the ReactomePA package (**D**–**G**). Significance denoted as ** *p* < 0.01.

Morphologically, BAT was characterised by a heterogenous mix of small, mitochondria rich lipid droplets and large lipid droplets, and adipocytes, indicative of large-scale whitening with thermoneutral housing from weaning (Figure 3A). Analysis of lipid droplet areas in BAT demonstrated a significant increase in both cold, and YM-178 treated rats (Figure 3B) with adipocyte size also increased in IWAT of cold-exposed rats (Figure 4A,B). Given the surprising increase in BAT and IWAT mass with cold exposure, we then carried out exploratory adipose tissue proteomics. This method, which quantifies the 30–40% most abundant proteins across samples, did not detect UCP1 in BAT, which is not unexpected given the chronic suppression of adaptive thermogenesis. We identified 175 differentially regulated proteins in BAT of cold-exposed rats (Figure 3C, Table 1 and Supplementary Data) including an increase in the mitochondrial citrate transporter protein (SLC25a1), glucose-6-phosphate dehydrogenase (G6PD), and the muscle isoforms of phosphoglycerate mutase (PGAM2) and creatine kinase (CKm).

Conversely, 137 proteins were differentially regulated in BAT of YM-178 treated animals (Figure 3C, Table 1 and Supplementary Data) with an upregulation of proteins involved in skeletal muscle physiology including myosin heavy chain 4 (MYH4), fast-twitch skeletal muscle isoforms troponin I2 (TNNI2) and calsequestrin 1 (CASQ1) in addition to proteins governing endothelial adhesion and vascular growth (PECAM1 and FBLN5). These changes occurred alongside a downregulation of the aldo-keto reductase family member proteins B15 (AKR1B15) and C3 (AKR1C3), mevalonate diphosphate decarboxylase (MVD), trans-2,3-enoyl-CoA reductase (TECR), acyl-CoA dehydrogenase short/branched chain (ACADSB), phosphate cytidylyltransferase 1, choline, alpha (PCYT1A) and 3-hydroxybutyrate dehydrogenase 1 (BDH1) proteins.

We then carried out functional analysis of the BAT proteome. The differentially regulated proteins in BAT of cold-exposed animals enriched GO terms involved ‘*glucose import*’, ‘*ATP-dependent helicase activity*’ and ‘*regulation of protein phosphorylation*’ whilst there was also an enrichment of nuclear related GO terms including ‘*histone deacetylation*’, ‘*nucleosomal DNA binding*’, ‘*nuclear chromatin*’ and the ‘*nucleosome*’ (Table 2 and Supplementary Data). Conversely, differentially regulated proteins in BAT of animals treated with YM-178 enriched GO terms including ‘*positive regulation of protein kinase B signalling*’, ‘*negative regulation of cellular carbohydrate metabolic process*’ and ‘*positive regulation of atpase activity*’ whilst there was also an enrichment of GO terms involved in both brown adipocyte and muscle biology including ‘*brown fat cell differentiation*’, ‘*skeletal muscle contraction*’ and ‘*regulation of muscle contraction*’ (Table 2 and Supplementary Data). Finally, using ReactomePA we show enriched pathways regulated by cold exposure (Figure 3D), and their interactions (Figure 3E), including ‘transcriptional regulation of RUNx1′, pathways involved in mitosis (i.e., G2/M transition), and the degradation of glycoproteins (i.e., CS/DS degradation). Conversely, YM-178 treatment enriched multiple pathways associated with proteolysis (i.e., eukaryotic translation initiation and formation of a pool of free 40S subunits) and RNA surveillance (i.e., nonsense mediated decay) (Figure 3F,G).

The substantial increase in IWAT of cold-exposed rats was associated with 116 differentially regulated proteins (Figure 4C, Table 3 and Supplementary Data) including the upregulation of lipid metabolic proteins such as fatty acid binding proteins 1 (FABP1) and 3 (FABP3), fatty acid synthase (FASN) and ATP citrate lyase (ACLY) and those involved in beta oxidation, including hydroxynacyl-CoA dehydrogenase (HADH) and acyl-CoA dehydrogenase long chain (ACADL). In addition, there was an upregulation of glycerol kinase (GK), pyruvate carboxylase (PC) and monocarboxylic acid transporter 1 (MCT1) that were accompanied by a downregulation of multiple proteins involved in mRNA processing and splicing (i.e., RNA binding motif protein 8B, RBM8B; dexd-Box helicase 39B, DDX39B and poly(u) binding splicing factor 60, PUF60).

YM-178 administration modulated 206 proteins in IWAT (Figure 4C, Table 3 and Supplementary Data) including an upregulation of multiple proteins involved in the nervous system including synuclein gamma (SNCG), neurolysin (NLN) and neuronal pas domain protein 4 (NPAS4). This was associated with an increase in proteins involved in lipid and cholesterol metabolism including carnitine palmitoyltransferase 1A (CPT1A), hormone-sensitive lipase (LIPE) and apolipoprotein C1 and M (APOC1 and APOM). Interestingly, YM-178 also induced an increase in multiple inflammatory proteins, including orosomucoid 1 (ORM1), complement C4A (C4A), S100 calcium binding protein A8 (S100A8) and S100 calcium binding protein B (S100B).

Differentially regulated proteins in IWAT of cold-exposed rats enriched GO terms involved in the ‘*DNA damage response*’, ‘*3-hydroxyacyl-coa dehydrogenase activity*’ and ‘*NAD+ binding*’ whilst YM-178 enriched inflammatory terms including the ‘*acute phase response*’ and ‘*structural constituent of ribosome*’ suggesting an effect of sympathetic activation on both the inflammatory system and protein synthesis (Table 4 and Supplementary Data). Finally, ReactomePA demonstrated enriched pathways (Figure 4D), and their interactions (Figure 4E) were associated with IWAT expansion including the ‘metabolism of amino acids and derivatives’ and ‘fatty acid metabolism’. Conversely, and similar to BAT, YM-178 treatment enriched multiple pathways associated with proteolysis (i.e., eukaryotic translation initiation and formation of a pool of free 40S subunits) and RNA surveillance (i.e., nonsense mediated decay) but to a larger degree (i.e., 23–27 proteins per term rather than 9–11; Figure 4F,G).

## 3. Discussion

Housing temperature, especially cold exposure, impacts on metabolic homeostasis as illustrated by the effects on BAT and ‘browning’ [3,15,16,17,18]. Little is known as to whether interventions considered to promote browning are effective in animals maintained at thermoneutrality [2,18]. Here we show that chronic exposure to a mild cold stimulus (i.e., standard housing temperature) drives weight gain and the deposition of large quantities of subcutaneous adipose tissue in obese rats rather than the activation of BAT and subsequent weight-loss as expected [1]. This effect is not seen in rats treated with the highly selective β3-agonist YM-178, suggesting sympathetic activation is not involved and it is a direct effect of ambient temperature. Taken together these findings are indicative of a novel mechanism whereby rats increase body weight and fat mass following chronic suppression of adaptive thermogenesis from weaning.

There is accumulating evidence that, in the absence of adaptive thermogenesis, other mechanisms compensate in order to maintain body temperature. For instance, in UCP1 k/o mice, a reduction in BAT thermogenesis leads to the recruitment of shivering thermogenesis in skeletal muscle [19]. Conversely, when shivering is impaired in Sarcolipin k/o mice, there is a compensatory increase in BAT activity [19]. In obese animals lacking BAT, there seems to be an entirely different homeostatic response to cold stress. Following BAT lipectomy, obese, cold-exposed rats gain weight and adipose tissue mass is nearly doubled [20] which mirrors our findings when BAT is chronically supressed. Furthermore, intermittent cold exposure (ICE, from 20 °C to 4 °C) over a period of days promotes weight gain and adiposity and this is associated with intermittent increases in energy intake [21]. Rats are also more susceptible to weight gain, and fat accumulation when reared in the cold (18 °C vs. 30 °C), an effect which persists when housed at a common temperature [22]. More recently, it was demonstrated that ICE increases weight gain, largely due to increases in adiposity, in both male and female mice housed at thermoneutrality and fed a diet high in fat [5]. Despite no effect on circulating NEFA or insulin concentrations, ICE increased lipid deposition in hepatocytes and circulating leptin whilst decreasing circulating triglycerides. Intriguingly, whilst ICE exacerbated diet-induced obesity it let to marked improvements in glucose homeostasis. This suggests that in human relevant conditions, i.e., thermoneutrality, both chronic and intermittent cold exposure may exacerbate weight gain through as yet unidentified mechanisms, but may, in the case of ICE, improve glucose homeostasis.

Whilst recent work on ICE demonstrated that cold-induced weight gain was dependant on energy intake, we did not see increased food intake suggesting another, unexplained mechanism, whereby cold induces adiposity. There is a plausible mechanism linking the gut to this phenotype which needs to be explored in future. Cold exposure drives intestinal growth, increased fatty acid absorption and paracellular permeability to nutrients [23,24]. The efficiency of energy utilisation is also sufficient to maintain core body temperature during acute cold exposure [25]. If the gut can grow, even during periods of energy restriction, and maximise absorption of energy to a degree that it sustains critical functions, and ultimately life, then it may be able to drive adiposity in a similar manner. Whilst the insulative effects of obesity are being debated [26,27,28] a model whereby rats, and potentially other rodents, deposit subcutaneous fat during exposure to cold (i.e., “store up nuts for the winter internally” [22]) would make sense, and be hugely beneficial from an evolutionary perspective given wild animals cannot simply increase energy intake during winter months.

Another important finding of this study is that the increase in weight gain and subcutaneous AT mass seen in cold-exposed rats was not associated with impaired metabolic parameters (i.e., fasting glucose and lipids) or any discernible adipose tissue dysfunction. Here, chronic exposure to a mild cold stimulus seemingly drives a phenotypically healthy expansion of subcutaneous AT. Using exploratory proteomics, we were able to elucidate processes in both BAT and IWAT associated with this expansion of AT mass. An increase in proteins that modulate metabolism, including those involved in glycolysis, the TCA cycle and lipogenesis, suggests that there is an increased metabolic flux in these depots which, ultimately, results in net lipogenesis. In BAT, this is associated with an enrichment of mitotic pathways (i.e., G2/M transition). The G2/M transition is a critical point in the cell cycle where, following DNA replication, the mitotic process begins, and cells separate into replicate daughter cells. Mitotic clonal expansion is essential for adipogenesis in 3T3-L1 adipocytes [29] and is one of the postulated mechanisms through which FTO regulates fat mass [30]. Further, enrichment of the ‘transcriptional regulation on RUNX1′ pathway, which regulates the white-to-brown/beige transition via CDK6 points towards the cell-cycle as a key mediator of increased BAT mass following cold exposure [31]. As such, an increase in mitosis of adipocyte pre-cursors, key changes in the cell cycle and subsequent adipogenesis may be partly driving increased BAT mass.

In IWAT, an upregulation of regulatory proteins involved in lipid metabolism (i.e., FABP1, FASN and ACLY) and beta oxidation (i.e., HADH and ACADL) in addition to GK, PC and MCT1 indicates an increased metabolic flux in IWAT. Importantly, however, adipocyte size in IWAT of cold-exposed rats only increased by ~17%. As such, we predict that this physiological expansion of IWAT is largely due to adipogenesis which ties in to the already established theory of ‘healthy adipose tissue expansion’ where growth of subcutaneous AT, through adipogenesis, protects from the adverse effects of an obesogenic diet [32].

We were also able to elucidate the impact of sympathetic activation in the absence of any change in housing temperature by administering YM-178. This highly selective β3-agonist increases BAT activity, whole body EE and induces ‘browning’ in humans [33,34,35,36]. YM-178 has a 20–200 higher agonist activity for both rat, and human bladder than other agonists and, despite differences in β-AR subtype expression between rats and humans, exhibits full agonistic activity in both species [37,38,39,40]. Recently, two landmark studies have demonstrated that YM-178 also improves glucose homeostasis and insulin sensitivity, improves β-cell function, reduces skeletal muscle triglyceride content and increases HDL cholesterol and adiponectin in overweight and obese prediabetic or insulin resistant humans [10,11]. However, these, and other studies show that human BAT activity and the response to either cold, or other thermogenic stimuli (i.e., YM-178) is highly heterogenous which is unsurprising given humans undergo seasonal BAT activation to varying degrees [41]. Our aim was to determine the efficacy of this treatment in a homogenous population where BAT had been supressed from early life. Under these conditions, YM-178 treatment had no impact on metabolic parameters or thermogenesis. It does, however, regulate pathways involved in protein synthesis in both BAT and, to a larger degree, IWAT. In the hippocampus, β3-AR agonism (by isoprenaline) drives ERK/MTOR dependent activation of eukaryotic initiation factor 4E and inhibition of the translation repressor 4E-BP whilst CL316, 243 and salbutamol drive skeletal muscle hypertrophy and protein synthesis in mice and humans, respectively [42,43,44]. This would suggest a novel role for YM-178 in regulating protein synthesis in adipose tissues which has yet to be examined. YM-178 also regulated proteins involved in the acute phase response and inflammation in both BAT and IWAT and increased lipid droplet size in BAT. It is unclear why, or how, β3-AR agonism would increase lipid droplet size in this setting however, when taken together, this suggests YM-178 treatment following chronic suppression of BAT may act negatively on AT driving inflammatory processes and lipid droplet/adipocyte hypertrophy. Whilst these differences may be species specific, they may also highlight potential effects of YM-178 if given to individuals lacking BAT, or who exhibit low responsiveness to thermogenic stimuli.

Whilst this study points to a novel, unexplained yet controversial adaptation to cold, it is important to remember the major differences in physiology and cellular processes in animals housed at different ambient temperatures [18]. These differences are likely exacerbated in animals who have been raised at thermoneutrality from weaning and it is not entirely unexpected that the physiological response to a commonly studied stimulus would be different in this scenario. It is clear that the early life environment, and early life stressors, impact adult physiology and susceptibility to disease, including obesity, and important adaptations during the developmental period may underlie the adaptations seen here. Despite aiming to mimic human physiology, this work cannot replicate the human in-utero, and birth environments, and there may be other key developmental changes in humans during this period that are not applicable here. Further, rearing temperature of rats is associated with changes in sympathoadrenal activity and rats are susceptible to obesity in a strain- and sex-dependent manner [22,45,46,47]. It will be important in future to determine if this effect is not only species specific, but also strain and sex specific as we look to untangle aspects of this biology to promote healthy expansion of adipose tissue in humans. Finally, whilst many of our OMICs hits are regulated by, or critical for the adaptation to cold or β3-AR agonism at standard housing temperatures this dataset provides a rich resource for further analysis of novel proteins associated with weight gain.

## 4. Conclusions

In summary, we show that chronic exposure to mild cold following chronic suppression of BAT drives weight gain and the deposition of large quantities of subcutaneous AT. Whilst the precise mechanism is not clear, this work points towards a novel response whereby animals increase body weight and fat mass in response to a reduction in ambient temperature. We propose that chronic suppression of BAT from weaning, using thermoneutral housing and an obesogenic diet, changes the physiological response to cold in the obese state. Instead, humanised animals deposit adipose tissue and gain weight, an effect not seen with YM-178, suggesting a direct effect of temperature, where an insulative mechanism is potentially recruited when a thermogenic response is absent.

## 5. Methods

### 5.1. Animals, Cold Exposure and YM-178 Treatment

All studies were approved by the University of Nottingham Animal Welfare and Ethical Review Board and were carried out in accordance with the UK Animals (Scientific Procedures) Act of 1986 (PPL no.). Eighteen male Sprague-Dawley rats aged 3 weeks were obtained from Charles River (Kent, UK) and housed immediately at thermoneutrality (c. 28 °C), on a high-fat diet (HFD; 45%, 824,018 SDS, Kent, UK), under a 12:12-h reverse light-dark cycle (lights off at 08:00). These conditions were chosen to closer mimic human physiology [3], minimise animal stress and maximise data quality and translatability [48]. At 12 weeks of age, all animals were randomised to either 4 weeks of standard housing temperature (20 °C, n = 6), daily YM-178 administration by oral gavage (28 °C + β3, at a clinically relevant dose of 0.75 mg/kg/day, n = 6) or HFD controls (28 °C, n = 6). Animals randomised to 20 °C were moved into an adjacent, climate-controlled room, for the 4-week period. In adherence to the National Centre for the Replacement, Refinement and Reduction of Animals in Research (NC3Rs), this experiment was run alongside our work looking at the effect of exercise training on ‘browning’ and utilised the same cohort of control HFD animals [2].

### 5.2. Metabolic Assessment and Tissue Collection

All animals were placed in an open-circuit calorimeter (CLAMS: Columbus Instruments, Linton Instrumentation, Palgrave, Norfolk, UK) for the final 48 h to enable the assessment of whole-body metabolism [49]. All animals were then weighed and fasted overnight prior to euthanasia by rising CO_2_ gradient. BAT, perivascular BAT (PVAT) from the thoracic aorta and IWAT were then rapidly dissected, weighed, snap-frozen in liquid nitrogen and stored at −80 °C for subsequent analysis.

### 5.3. Histology

Adipose tissue histology was performed as previously described [8]. Briefly, BAT and IWAT were fixed in formalin, embedded in wax using an Excelsior ES processor (Thermo-Fisher, Runcorn, Cheshire, UK) and stained using haematoxylin and eosin (Sigma-Aldrich, Gillingham, Dorset, UK). Between 3 and 5 random sections were then imaged at 10x with an Olympus BX40 microscope and adipocyte size was quantified using Adiposoft [50].

### 5.4. Gene Expression Analysis

Total RNA was extracted from each fat depot using the RNeasy Plus Micro extraction kit (Qiagen, West Sussex, UK) using an adapted version of the single step acidified phenol-chloroform method. RT-qPCR was carried out as previously described using rat-specific oligonucleotide primers (Sigma-Aldrich, Missouri, MO, USA) or FAM-MGB Taqman probes [49]. Gene expression was determined using the GeNorm algorithm against two selected reference genes; *RPL19:RPL13a* in *BAT* and IWAT (stability value M = 0.163 in BAT and 0.383 in IWAT) and RPL19:HPRT1 in PVAT (stability value M = 0.285).

### 5.5. Serum Analysis

Glucose (GAGO-20, Sigma-Aldrich, Gillingham, UK), triglycerides (LabAssay Trigylceride, Wako, Neuss, Germany), non-esterified fatty acids (NEFA-HR(2), Wako, Neuss, Germany), insulin (80-INSRT-E01, Alpco, Salem, NH, USA) and leptin (EZRL-83K, Merck, Darmstadt, Germany) were measured as previously described following the manufacturer’s instructions [8].

### 5.6. Adipose Tissue Proteomics

Protein extraction, clean up and trypsinisation was performed on 50–100 mg of frozen tissue (n = 4/group) was homogenised in 500 μL CellLytic MT cell lysis buffer (Sigma, C3228) prior to removal of lipid and other contaminants using the ReadyPrep 2D cleanup Kit (Bio-Rad, Munich, Germany, 1632130) [49]. Samples were then subjected to reduction, alkylation and overnight trypsinisation following which they were dried down at 60 °C for 4 h and stored at 80 °C before resuspension in LCMS grade 5% acetonitrile in 0.1% formic acid for subsequent analysis. Analysis by mass spectrometry was carried out on a SCIEX TripleTOF 6600 instrument [51] with samples analysed in both SWATH (Data Independent Acquisition) and IDA (Information Dependent Acquisition) modes for quantitation and spectral library generation respectively. IDA data was searched together using ProteinPilot 5.0.2 to generate a spectral library and SWATH data was analysed using Sciex OneOmics software [52] extracted against the locally generated library as described previously [49].

### 5.7. Statistical Analysis

Statistical analysis was performed in GraphPad Prism version 8.0 (GraphPad Software, San Diego, CA, USA). Data are expressed as mean ± SEM with details of specific statistical tests in figure legends. Despite prior use of the control group to understand the impact of exercise training, only the groups included in this paper were utilised for analyses [8]. Functional analysis of the proteome was performed using the Advaita Bioinformatic iPathwayGuide software (www.advaitabio.com/ipathwayguide.html accessed on 30 December 2021) (fold change ± 0.5 and confidence score cut-off of 0.75). Significantly impacted biological processes and molecular functions were analysed in the context of the Gene Ontology Consortium database (November 2017) [53]. The Elim pruning method, which removes genes mapped to a significant GO term from more general (higher level) GO terms, was used to overcome the limitation of errors introduced by considering genes multiple times [54]. Pathway analysis was carried out using the ReactomePA package on R Studio (version 3.6.2) with a false discovery rate of <0.1.

## Figures and Tables

**Table 1:** Full list of differentially regulated proteins in brown adipose tissue.

Gene ID	Gene Name	Logfc	Adjpv
**20** **°** **C**			
80754	Rabep2	3.69	6.35 × 10^−5^
114122	Vcan	2.74	0.000269
292073	Galns	−2.75	0.00027
64012	Rad50	1.50	0.000288
171139	Timm9	−1.94	0.00037
295088	Gmps	0.67	0.000396
81716	Ggcx	1.44	0.000639
25622	Ptpn11	−2.47	0.000975
289590	Ociad1	−1.41	0.000988
29384	H2afy	−1.15	0.001077
**28 °C + B3**			
116689	Ptpn6	−3.56	0.000154
25650	Atp1b1	−1.40	0.000303
59108	Mb	2.71	0.000548
306262	Btd	2.72	0.000735
501167	Gmppa	1.58	0.000934
64528	Golga2	0.98	0.001723
287633	Lrrc59	−0.62	0.002902
81726	Mvd	−3.82	0.004012
363425	Cav2	−2.41	0.004547
114559	Arhgef7	−1.54	0.005187

**Table 2:** Full list of GO terms enriched in brown adipose tissue.

Go ID	Go Name	Count DE	Count All	Pv_Elim
**20 °C**				
**Biological Process**				
GO:0000122	negative regulation of transcription from RNA polymerase II promoter	14	40	0.008
GO:0003006	developmental process involved in reproduction	20	68	0.0146
GO:0071786	endoplasmic reticulum tubular network organization	3	4	0.0209
GO:0019098	reproductive behavior	3	4	0.0209
GO:0006544	glycine metabolic process	3	4	0.0209
GO:0016226	iron-sulfur cluster assembly	3	4	0.0209
GO:0090068	positive regulation of cell cycle process	7	17	0.0231
GO:0046323	glucose import	6	14	0.0285
GO:0001932	regulation of protein phosphorylation	32	129	0.0316
GO:0098969	neurotransmitter receptor transport to postsynaptic membrane	2	2	0.0333
**Molecular Function**				
GO:0000980	RNA polymerase II distal enhancer sequence-specific DNA binding	4	5	0.0046
GO:0030984	kininogen binding	3	3	0.006
GO:0031492	nucleosomal DNA binding	4	6	0.0119
GO:0001846	opsonin binding	3	4	0.0207
GO:0005212	structural constituent of eye lens	3	4	0.0207
GO:0016634	oxidoreductase activity, acting on the CH-CH group of donors, oxygen as acceptor	3	4	0.0207
GO:0016831	carboxy-lyase activity	5	10	0.022
GO:0016746	transferase activity, transferring acyl groups	9	25	0.0259
GO:0004616	phosphogluconate dehydrogenase (decarboxylating) activity	2	2	0.0331
GO:0008484	sulfuric ester hydrolase activity	2	2	0.0331
**Cellular Component**				
GO:0042582	azurophil granule	4	4	0.0011
GO:0000790	nuclear chromatin	12	27	0.0014
GO:0031616	spindle pole centrosome	3	4	0.0209
GO:0000786	nucleosome	5	10	0.0223
GO:0042719	mitochondrial intermembrane space protein transporter complex	2	2	0.0333
GO:0001740	Barr body	2	2	0.0333
GO:0072687	meiotic spindle	2	2	0.0333
GO:0034751	aryl hydrocarbon receptor complex	2	2	0.0333
GO:0043196	varicosity	2	2	0.0333
GO:0001931	uropod	3	5	0.0453
**28 °C + B3**				
**Biological Process**				
GO:0003009	skeletal muscle contraction	5	7	0.0015
GO:0051897	positive regulation of protein kinase B signaling	6	11	0.0034
GO:0010677	negative regulation of cellular carbohydrate metabolic process	3	3	0.0039
GO:1901896	positive regulation of calcium-transporting ATPase activity	3	3	0.0039
GO:0032781	positive regulation of ATPase activity	9	15	0.0055
GO:0050873	brown fat cell differentiation	3	4	0.0138
GO:0006937	regulation of muscle contraction	8	22	0.0147
GO:0071560	cellular response to transforming growth factor beta stimulus	8	22	0.0147
GO:0060048	cardiac muscle contraction	6	15	0.0209
GO:0048193	Golgi vesicle transport	12	42	0.024
**Molecular Function**				
GO:0035259	glucocorticoid receptor binding	3	4	0.013
GO:0001671	ATPase activator activity	3	4	0.013
GO:0008134	transcription factor binding	15	55	0.016
GO:0050431	transforming growth factor beta binding	2	2	0.024
GO:0031730	CCR5 chemokine receptor binding	2	2	0.024
GO:0031014	troponin T binding	2	2	0.024
GO:0005044	scavenger receptor activity	3	5	0.029
GO:0019905	syntaxin binding	4	9	0.037
GO:0016779	nucleotidyltransferase activity	4	9	0.037
GO:0004888	transmembrane signaling receptor activity	4	9	0.037
**Cellular Component**				
GO:0005887	integral component of plasma membrane	12	32	0.002
GO:0030134	COPII-coated ER to Golgi transport vesicle	5	9	0.0067
GO:0005861	troponin complex	2	2	0.0245
GO:0001741	XY body	2	2	0.0245
GO:0043596	nuclear replication fork	3	5	0.0299
GO:0044295	axonal growth cone	3	5	0.0299
GO:0016459	myosin complex	5	13	0.0399

**Table 3:** Full list of differentially regulated proteins in white adipose tissue.

Gene ID	Gene Name	Logfc	Adjpv
**20 °C**			
117028	Bin1	−2.79	8.97 × 10^−6^
304290	Kdelr2	−2.68	4.47 × 10^−5^
24667	Ppm1b	−2.65	0.000108
84114	Agps	−1.35	0.000125
84401	Puf60	−2.94	0.000753
300983	Abhd14b	0.94	0.000791
29218	Rcn2	−2.40	0.000854
290028	Osgep	−0.94	0.002064
24230	Tspo	−2.34	0.00248
171452	Rab3il1	−2.15	0.00629
**28 °C + B3**			
83730	Vamp8	−3.75	6.93 × 10^−5^
29521	Scamp1	1.51	0.000111
25116	Hsd11b1	0.92	0.000382
117045	Eif4e	−0.69	0.000942
25342	Oxtr	1.79	0.001104
298566	C1qa	0.85	0.001124
445268	Ufc1	−0.65	0.001133
78947	Gcs1	0.61	0.00204
266734	Npas4	0.87	0.004234
246303	Serbp1	0.78	0.004516

**Table 4:** Full list of GO terms enriched in white adipose tissue.

Go ID	Go Name	Count DE	Count All	Pv_Elim
**20 °C**				
**Biological Process**				
GO:0030330	DNA damage response, signal transduction by p53 class mediator	5	6	0.0022
GO:0048711	positive regulation of astrocyte differentiation	3	3	0.0023
GO:0071498	cellular response to fluid shear stress	3	3	0.0023
GO:0032780	negative regulation of ATPase activity	3	3	0.0023
GO:0051607	defense response to virus	5	9	0.003
GO:0001822	kidney development	11	35	0.0036
GO:0050731	positive regulation of peptidyl-tyrosine phosphorylation	7	17	0.0037
GO:0002244	hematopoietic progenitor cell differentiation	3	4	0.0081
GO:0045577	regulation of B cell differentiation	3	4	0.0081
GO:0042130	negative regulation of T cell proliferation	3	4	0.0081
**Molecular Function**				
GO:0051287	NAD binding	11	31	0.0013
GO:0008144	drug binding	9	29	0.0101
GO:0005001	transmembrane receptor protein tyrosine phosphatase activity	2	2	0.0178
GO:0005521	lamin binding	3	5	0.0191
GO:0042393	histone binding	5	13	0.021
GO:0033613	activating transcription factor binding	3	6	0.0345
GO:0003857	3-hydroxyacyl-CoA dehydrogenase activity	3	6	0.0345
GO:0045296	cadherin binding	22	113	0.0365
GO:0004028	3-chloroallyl aldehyde dehydrogenase activity	2	3	0.0486
GO:0071933	Arp2/3 complex binding	2	3	0.0486
**Cellular Component**				
GO:0005884	actin filament	9	24	0.0021
GO:0032993	protein-DNA complex	5	11	0.0088
GO:0002102	podosome	7	14	0.013
GO:0016607	nuclear speck	8	26	0.0146
GO:0031209	SCAR complex	2	2	0.0172
GO:0042611	MHC protein complex	2	2	0.0172
GO:0005687	U4 snRNP	2	2	0.0172
GO:0005856	cytoskeleton	49	238	0.0284
GO:0030054	cell junction	41	216	0.0291
GO:0005681	spliceosomal complex	9	22	0.0311
**28 °C + B3**				
**Biological Process**				
GO:0006953	acute-phase response	7	12	0.0035
GO:0000381	regulation of alternative mRNA splicing, via spliceosome	7	12	0.0035
GO:0034113	heterotypic cell-cell adhesion	8	12	0.0061
GO:0070528	protein kinase C signaling	4	5	0.0063
GO:0015671	oxygen transport	4	5	0.0063
GO:1901741	positive regulation of myoblast fusion	3	3	0.0077
GO:0070934	CRD-mediated mRNA stabilization	3	3	0.0077
GO:0007566	embryo implantation	6	12	0.0179
GO:0040007	growth	29	103	0.0209
GO:0071345	cellular response to cytokine stimulus	25	86	0.0211
**Molecular Function**				
GO:0003735	structural constituent of ribosome	24	62	0.00037
GO:0005344	oxygen carrier activity	4	5	0.00657
GO:0003730	mRNA 3’-UTR binding	8	18	0.01548
GO:0003682	chromatin binding	10	25	0.01623
GO:0140097	catalytic activity, acting on DNA	5	9	0.01908
GO:0042162	telomeric DNA binding	3	4	0.02688
GO:0045294	alpha-catenin binding	3	4	0.02688
GO:0004527	exonuclease activity	3	4	0.02688
GO:0003723	RNA binding	73	281	0.02734
GO:0019825	oxygen binding	4	7	0.03278
**Cellular Component**				
GO:0022625	cytosolic large ribosomal subunit	15	30	0.00018
GO:0016323	basolateral plasma membrane	14	32	0.00164
GO:0005833	hemoglobin complex	3	3	0.00782
GO:0005903	brush border	10	23	0.00812
GO:0030864	cortical actin cytoskeleton	9	20	0.00918
GO:0016327	apicolateral plasma membrane	3	4	0.02666
GO:0044451	nucleoplasm part	18	58	0.0269
GO:0005637	nuclear inner membrane	5	10	0.03167
GO:0035770	ribonucleoprotein granule	11	32	0.03804
GO:0097225	sperm midpiece	2	2	0.03953

## Data Availability

The datasets used and analysed during the current study are available from the corresponding author on reasonable request.

## Supplementary Materials

The following supporting information can be downloaded at: https://www.mdpi.com/article/10.3390/ijms23031869/s1.
