# Supplementary material for: Cold Exposure Drives Weight Gain and Adiposity following Chronic Suppression of Brown Adipose Tissue
Source: Int J Mol Sci. 2022 Feb 7;23(3):1869. doi: 10.3390/ijms23031869 (PMC8836787; doi:10.3390/ijms23031869)
Supplement: Supplementary file 1 [file ijms-23-01869-s001.zip › ijms-1478698-supplementary.pdf]

International Journal of *Molecular Sciences*

MDPI

Supplementary data

Article

Cold Exposure Drives Weight Gain and Adiposity following Chronic Suppression of Brown Adipose Tissue

Peter Aldiss [1,2,*], Jo E. Lewis [3], Irene Lupini [4], Ian Bloor [1], Ramyar Chavoshinejad [1], David J. Boocock [5], Amanda K. Miles [5], Francis J. P. Ebling [3], Helen Budge [1] and Michael E. Symonds [1,6,*]

[1] Academic Unit of Population and Lifespan Sciences, Centre for Perinatal Research, School of Medicine, University of Nottingham, Nottingham NG7 2UH , UK; ian.bloor@nottingham.ac.uk (I.B.); ramyar.chavoshinejad@nottingham.ac.uk (R.C.); helen.budge@nottingham.ac.uk (H.B.)
[2] Section for Nutrient and Metabolite Sensing, The Novo Nordisk Foundation Center for Basic Metabolic Research, University of Copenhagen, 2200 Copenhagen, Denmark
[3] Queen's Medical Centre, School of Life Sciences, University of Nottingham, Nottingham NG11 8N, UK; jl2033@medschl.cam.ac.uk (J.E.L.); fran.ebling@nottingham.ac.uk (F.J.P.E.)
[4] School of Biosciences and Veterinary Medicine, University of Camerino, 62032 Camerino, Italy; Irene.lupini@studenti.unicam.it
[5] John van Geest Cancer Research Centre, Nottingham Trent University, Nottingham NG11 8N, UK; david.boocock@ntu.ac.uk (D.B.); amanda.miles@ntu.ac.uk (A.K.M.)
[6] Nottingham Digestive Disease Centre Biomedical Research Center, School of Medicine, University of Nottingham, Nottingham NG11 8N, UK
* Correspondence: Peter.aldiss@sund.ku.dk(P.A.); michael.symonds@nottingham.ac.uk (M.E.S.)

Table S1. Full list of differentially regulated proteins in BAT.

GeneID	Gene name	logfc	adjpv
**20°C**			
80754	Rabep2	3.69	6.35E-05
114122	Vcan	2.74	0.000269
292073	Galns	-2.75	0.00027
64012	Rad50	1.50	0.000288
171139	Timm9	-1.94	0.00037
295088	Gmps	0.67	0.000396
81716	Ggcx	1.44	0.000639
25622	Ptpn11	-2.47	0.000975
289590	Ociad1	-1.41	0.000988
29384	H2afy	-1.15	0.001077
302669	Ca5b	1.18	0.001237
499991	Steap4	-3.88	0.00228
315265	Twf1	-1.54	0.00248
25283	Gclc	2.57	0.002571
84474	Ddx1	2.23	0.00326
94342	Bag6	-1.26	0.0036
89827	Ddx39a	1.26	0.00397
64679	Tgm4	0.95	0.00616
64517	Thop1	1.25	0.006186
29743	Slc25a1	1.10	0.006388
64630	Snap23	-0.57	0.006562
79449	Rpl21	-2.43	0.007106
57341	Parva	-1.36	0.009375

305178	Hnrnpdl	-0.75	0.00987
85274	Prdx4	-3.77	0.010514
259275	Ostf1	0.84	0.010579
29688	Minpp1	-2.07	0.013155
445268	Ufc1	-2.04	0.014068
24959	Pgam2	1.80	0.014105
361613	Ppme1	2.03	0.014231
307039	Rab18	-0.80	0.015098
304024	Cpox	2.64	0.017902
100909840	LOC100909840	-0.60	0.017981
81781	Snrpn	-0.51	0.018985
690131	Hist2h2aa2	-0.61	0.019641
497198	Impact	-1.80	0.020479
307905	Usp10	-3.13	0.020618
499839	RGD1564664	-2.70	0.021669
25010	Scgb2a1	-3.44	0.022101
308650	Hnrnph2	2.88	0.022881
310695	Kirrel1	2.62	0.025104
363013	Tmem123	2.59	0.026566
170751	Xpnpep1	3.29	0.02881
100360180	Pgd	0.53	0.028867
25420	Cryab	0.90	0.029983
311422	Itpa	0.78	0.032824
29676	Psmb3	-1.65	0.033466
29734	Hspa13	0.88	0.034403
25537	Rock2	2.20	0.036381
305497	Cobl	2.18	0.03795
290640	Map1s	-3.42	0.042638
29637	Hmgcs1	4.22	0.042914
308384	Sae1	-2.63	0.043105
300757	Hexa	1.15	0.046193
83764	Flot2	1.95	0.048583
103689947	LOC103689947	-0.50	0.055615
25425	Ctsh	0.58	0.055862
140868	Fabp5	0.63	0.057439
50681	Acox1	-1.28	0.058114
295692	Nup35	0.61	0.063838
314648	Ncln	-4.13	0.063902
64157	Ddah1	1.36	0.066755
81666	Gnaq	-1.16	0.068826
192360	Eml2	0.81	0.07351
303518	Smarce1	4.10	0.073866
307503	Etf1	-1.94	0.074281
192235	Hyou1	1.79	0.074523
287828	Jpt1	0.79	0.074615
297566	Atp6v1e1	0.62	0.074767
84357	Sh3kbp1	1.45	0.075562
117104	Ppp2r2a	0.71	0.075654
192249	Ehd3	1.65	0.075682
58815	Glrx3	-3.87	0.077933
117028	Bin1	-0.89	0.082843

59303	Tmem33	1.06	0.085507
29425	Psmb5	2.58	0.08587
24157	Acaa1a	-0.72	0.089194
306283	Anxa8	-0.66	0.089575
362015	Ampd2	1.19	0.090078
691657	Crip1	1.32	0.094208
294673	Hexb	-2.92	0.102142
79210	Fstl1	1.71	0.102491
680522	Hist1h1b	1.38	0.103409
683788	Fscn1	0.53	0.106285
64032	Ctgf	-0.50	0.106913
100361558	LOC100361558	1.24	0.107837
114766	Phb2	-1.11	0.108469
29254	Mgll	0.74	0.112464
287042	Nubp1	1.49	0.114342
64203	Bcat2	-1.08	0.114377
102550391	LOC102550391	-0.71	0.114543
361532	Sirt2	1.80	0.115482
361635	LOC361635	-1.12	0.120085
157074	Sdha	0.55	0.122822
64347	Sncg	0.94	0.132378
361884	Mccc2	-0.58	0.13291
298609	Efhd2	1.97	0.132936
89841	Pcyt2	2.41	0.133672
25277	Mfge8	1.81	0.133882
27139	Rps26	-0.91	0.134129
83781	Lgals3	0.70	0.13543
299027	Eif2s3	0.53	0.136792
361092	Stk24	1.58	0.138388
360820	Pxn	2.40	0.138502
29153	Capn1	1.18	0.140233
287125	Nubp2	-1.48	0.142311
89825	Nap1l1	-2.20	0.144302
85492	Psmb7	2.27	0.147263
63938	Hibadh	-0.71	0.152486
60373	Nop58	2.02	0.153349
64538	Ilkap	-1.24	0.153505
301252	Hsp90ab1	-0.69	0.154211
25177	Fhl1	1.57	0.154475
252928	Timm13	-1.25	0.154815
54321	Cnn3	2.50	0.155197
362634	C1qc	-1.45	0.155978
300981	Acy1	-0.60	0.15657
117041	Nln	-2.71	0.161382
24655	Plcd1	1.36	0.16585
25344	Phb	-0.91	0.166831
300968	Uba5	2.00	0.167161
25380	Anxa1	0.91	0.170097
315707	Csk	-3.52	0.170238
282827	Aip	0.99	0.171773
114123	Sardh	0.82	0.172193

114113	Pafah1b3	-3.72	0.173067
108348260	LOC108348260	-1.77	0.17431
301618	Ppp1r7	2.07	0.176101
81661	Gmfb	-3.15	0.179404
361927	Fxr1	1.22	0.182166
315664	Kdelc2	-3.19	0.182306
297893	Hdac1	1.93	0.185012
117130	Grifin	-1.17	0.187328
60466	Stx7	1.26	0.191458
313770	Mxra8	1.83	0.191508
29318	Ddt	-0.85	0.191745
291081	Tubb2b	0.53	0.195288
314644	Dohh	-2.00	0.196706
24437	H1f0	5.60	0.197351
24787	Sod2	-0.79	0.197772
29332	Stmn1	-3.43	0.198118
60384	Copb2	-1.96	0.198454
501232	Cesl1	1.17	0.198671
29681	C1qbp	-0.75	0.199398
362282	Pck1	-0.86	0.199468
298370	Txndc12	-0.69	0.199832
298441	Nasp	-1.22	0.200112
24377	G6pd	0.71	0.201778
317259	Nono	-0.71	0.202927
58835	Phgdh	0.60	0.203119
297699	Strap	3.00	0.204399
83572	Pafah1b1	-0.64	0.204626
25581	Psmc2	-2.54	0.205136
64201	Slc25a11	-4.80	0.207437
25643	Gnai3	2.86	0.207957
286896	Sgpl1	1.24	0.209271
246298	Retsat	0.60	0.209863
691947	Eif3j	1.23	0.211981
171155	Hadhb	-0.80	0.212642
64665	Flot1	1.11	0.216866
79248	Abca2	0.82	0.218028
293719	Ubxn1	1.55	0.220695
65204	Cnn1	5.54	0.223415
24265	Ckm	2.78	0.223581
287876	Actg1	0.54	0.229322
117103	Rab8a	2.01	0.229323
299201	Dlst	-0.60	0.230346
140934	Elp1	0.69	0.23234
313047	Yars	1.50	0.233809
171133	Gcsh	-1.65	0.23408
140931	Hnrnph1	-1.11	0.235857
290401	Esd	1.41	0.238483
66028	Arl6ip5	3.95	0.238743
310635	Arhgef2	-2.38	0.239455
25697	Ctsl	1.23	0.23982
**28°C+B3**			

116689	Ptpn6	-3.56	0.000154
25650	Atp1b1	-1.40	0.000303
59108	Mb	2.71	0.000548
306262	Btd	2.72	0.000735
501167	Gmppa	1.58	0.000934
64528	Golga2	0.98	0.001723
287633	Lrrc59	-0.62	0.002902
81726	Mvd	-3.82	0.004012
363425	Cav2	-2.41	0.004547
114559	Arhgef7	-1.54	0.005187
25737	Pcna	1.41	0.005462
171516	Akr1c3	-3.61	0.006199
311328	Rmdn3	-2.54	0.006373
312398	Smarcad1	-2.97	0.006491
29583	Pecam1	3.43	0.006613
25491	Nes	-1.35	0.008957
25106	Rgn	1.79	0.009092
117099	Bdh1	-1.76	0.009659
100364457	LOC100364457	0.98	0.010813
681429	Rps27l	1.68	0.011212
24439	Hagh	4.52	0.01207
365377	Trim72	-2.27	0.012326
266605	Dcps	0.74	0.012836
113922	Selenof	-1.84	0.013927
81922	Sh3gl1	-0.70	0.015702
116463	Akr1b7	-4.12	0.016705
309593	Gnl1	1.21	0.017056
360543	Myh4	3.19	0.021934
294853	Krt18	-4.53	0.022348
291434	Rpl17	0.57	0.024257
266759	Hspa4	1.93	0.025477
29671	Psma4	2.04	0.027228
100910732	LOC100910732	-4.65	0.02735
29437	Acta1	2.41	0.031493
295703	Serping1	1.24	0.033653
25618	Acadsb	-2.31	0.034211
81651	Cspg4	1.02	0.034735
124461	Pacsin2	-1.11	0.036395
260321	Fkbp4	-1.82	0.039467
364064	Pycr2	1.40	0.040704
681913	Gstz1	1.68	0.041137
170845	Ndel1	-5.37	0.041759
116547	S100a8	-1.40	0.045185
366734	Bag5	-3.35	0.04572
296570	Edf1	-2.35	0.046418
81520	Marcksl1	-0.88	0.046841
140544	Pcyt1a	-2.97	0.047611
501203	Myl12a	0.52	0.049269
83712	Rbbp7	-2.70	0.051168
25371	Adprh	-1.34	0.052742
498545	Tsc22d1	-2.36	0.056873

108348287	LOC108348287	1.58	0.05858
29435	Ssr4	2.47	0.061261
25484	Myo1e	-2.31	0.062683
287191	Rars	3.19	0.067376
81815	Tpp2	-2.81	0.069101
84379	Rab6a	0.51	0.070121
59114	Slc9a3r1	1.19	0.078356
140673	Napa	1.34	0.07892
313647	Hp1bp3	6.26	0.079022
298098	Pole3	0.74	0.079152
362154	Zc3h15	-1.20	0.08213
25338	Ninj1	-2.78	0.082898
25621	Cd81	-0.60	0.084793
81761	Rnpep	1.43	0.086835
361207	Tmed9	2.00	0.089368
114861	Scpep1	0.83	0.089463
65028	Dnaja1	-1.01	0.089869
59107	Ltbp1	-2.60	0.092309
292023	Aars	-0.73	0.092548
29285	Rps15	-2.97	0.096805
29283	Rpl29	0.70	0.097067
24946	F9	1.73	0.098183
191576	Tecr	-2.74	0.098231
296320	Ctnnbl1	-1.42	0.10886
289456	Hsd17b11	0.92	0.110043
686019	Casq1	2.72	0.110524
100359982	Mpc2	-5.03	0.113167
29158	Fbln5	2.97	0.114676
314730	Ikbip	-1.76	0.116982
246325	Kcnh8	0.84	0.117573
58945	Dynll1	5.47	0.118062
408248	Psma3l	-0.90	0.119604
361730	Tkfc	-2.20	0.12089
259246	LOC259246	-0.81	0.124788
360629	Nt5c3b	-1.17	0.126636
684527	Crtc1	2.24	0.127565
171063	Gtf3c1	-1.91	0.130115
94266	Rps27	0.77	0.136434
288022	Ccdc50	-2.18	0.143163
192269	Sub1	2.53	0.143954
362173	Caprin1	-3.02	0.144588
299923	Ndrg1	-1.10	0.148883
114023	Copb1	1.04	0.155732
116482	Sacm1l	2.79	0.155855
25291	Anxa3	-0.51	0.159588
117268	Khdrbs1	-3.10	0.165319
619580	Ctps2	-0.62	0.166527
94174	Tinagl1	-3.84	0.166646
298566	C1qa	1.55	0.171369
25475	Lgals5	2.84	0.171861
313200	Hsdl2	0.77	0.17566

25073	Scarb1	1.21	0.176283
296851	Pon2	-0.64	0.179464
113956	Pecr	2.01	0.181245
308796	Mesd	-1.28	0.183137
289144	Cacybp	-0.55	0.183169
171105	Lnpep	-1.78	0.183999
94197	Rab14	2.32	0.188069
64367	Ppib	-0.58	0.191775
25125	Stat3	-2.20	0.19204
245955	Lgals3bp	-1.76	0.194232
301442	Sumo1	-0.98	0.194283
171562	Ero1a	0.75	0.196251
81827	Psmc5	-2.94	0.199308
25614	Ptk2	1.49	0.201321
290500	Ggact	2.29	0.204519
93646	Sec31a	1.75	0.211889
54318	Eif2s1	-2.50	0.216204
296554	Tubb4b	-0.53	0.216562
266760	Nalcn	-3.14	0.217229
81763	Rpl5	2.72	0.222544
84401	Puf60	-2.17	0.224326
29389	Tnni2	2.61	0.224664
117152	Cand1	0.74	0.224995
140922	Txnl1	-0.68	0.231544
24614	Orm1	0.85	0.233416
117272	Prpsap2	1.91	0.233601
338401	Crip2	1.21	0.237137
64196	Safb	0.53	0.238084
116666	Lman1	-0.57	0.242846
64667	Sgta	-0.88	0.242935
81776	Rps24	1.18	0.24314
84471	Snx1	-2.20	0.243648
64198	Pmpcb	-1.07	0.246348
171145	Eif2b3	2.20	0.248176
81653	Dbn1	-1.67	0.248852

Table S2. Full list of GO terms enriched in BAT.

Goid	Goname	Countde	Countall	Pv_Elim
	**20°C**			
	**Biological Process**			
GO:0000122	negative regulation of transcription from RNA polymerase II promoter	14	40	0.008
GO:0003006	developmental process involved in reproduction	20	68	0.0146
GO:0071786	endoplasmic reticulum tubular network organization	3	4	0.0209
GO:0019098	reproductive behavior	3	4	0.0209
GO:0006544	glycine metabolic process	3	4	0.0209
GO:0016226	iron-sulfur cluster assembly	3	4	0.0209
GO:0090068	positive regulation of cell cycle process	7	17	0.0231

GO:0046323	glucose import	6	14	0.0285
GO:0001932	regulation of protein phosphorylation	32	129	0.0316
GO:0098969	neurotransmitter receptor transport to postsynaptic membrane	2	2	0.0333
GO:1903044	protein localization to membrane raft	2	2	0.0333
GO:0032962	positive regulation of inositol trisphosphate biosynthetic process	2	2	0.0333
GO:0032986	protein-DNA complex disassembly	2	2	0.0333
GO:0050884	neuromuscular process controlling posture	2	2	0.0333
GO:0051482	positive regulation of cytosolic calcium ion concentration involved in phospholipase C-activating G-protein coupled signaling pathway	2	2	0.0333
GO:1902667	regulation of axon guidance	2	2	0.0333
GO:0031498	chromatin disassembly	2	2	0.0333
GO:0019322	pentose biosynthetic process	2	2	0.0333
GO:0060766	negative regulation of androgen receptor signaling pathway	2	2	0.0333
GO:0060338	regulation of type I interferon-mediated signaling pathway	2	2	0.0333
GO:1990592	protein K69-linked ufmylation	2	2	0.0333
GO:0003085	negative regulation of systemic arterial blood pressure	2	2	0.0333
GO:2000757	negative regulation of peptidyl-lysine acetylation	2	2	0.0333
GO:1900138	negative regulation of phospholipase A2 activity	2	2	0.0333
GO:0045039	protein import into mitochondrial inner membrane	2	2	0.0333
GO:0006689	ganglioside catabolic process	2	2	0.0333
GO:2000322	regulation of glucocorticoid receptor signaling pathway	2	2	0.0333
GO:0071169	establishment of protein localization to chromatin	2	2	0.0333
GO:0061582	intestinal epithelial cell migration	2	2	0.0333
GO:0009051	pentose-phosphate shunt, oxidative branch	2	2	0.0333
GO:0070193	synaptonemal complex organization	2	2	0.0333
GO:0070192	chromosome organization involved in meiotic cell cycle	2	2	0.0333
GO:0008277	regulation of G-protein coupled receptor protein signaling pathway	5	11	0.0349
GO:0006334	nucleosome assembly	6	15	0.0405
GO:0046578	regulation of Ras protein signal transduction	6	15	0.0405
GO:0010501	RNA secondary structure unwinding	4	8	0.0413
GO:0007626	locomotory behavior	7	19	0.043
GO:0043901	negative regulation of multi-organism process	7	19	0.043
GO:0072321	chaperone-mediated protein transport	3	5	0.0452
GO:0071468	cellular response to acidic pH	3	5	0.0452
GO:0051785	positive regulation of nuclear division	3	5	0.0452
GO:0045599	negative regulation of fat cell differentiation	3	5	0.0452
GO:0031647	regulation of protein stability	15	53	0.0455
GO:1901653	cellular response to peptide	16	58	0.0492
	**Molecular Function**			
GO:0000980	RNA polymerase II distal enhancer sequence-specific DNA binding	4	5	0.0046

GO:0030984	kininogen binding	3	3	0.006
GO:0031492	nucleosomal DNA binding	4	6	0.0119
GO:0001846	opsonin binding	3	4	0.0207
GO:0005212	structural constituent of eye lens	3	4	0.0207
GO:0016634	oxidoreductase activity, acting on the CH-CH group of donors, oxygen as acceptor	3	4	0.0207
GO:0016831	carboxy-lyase activity	5	10	0.022
GO:0016746	transferase activity, transferring acyl groups	9	25	0.0259
GO:0004616	phosphogluconate dehydrogenase (decarboxylating) activity	2	2	0.0331
GO:0008484	sulfuric ester hydrolase activity	2	2	0.0331
GO:0004563	beta-N-acetylhexosaminidase activity	2	2	0.0331
GO:0017136	NAD-dependent histone deacetylase activity	2	2	0.0331
GO:0102148	N-acetyl-beta-D-galactosaminidase activity	2	2	0.0331
GO:0008026	ATP-dependent helicase activity	5	11	0.0344
GO:0005546	phosphatidylinositol-4,5-bisphosphate binding	4	8	0.0409
GO:0004527	exonuclease activity	3	5	0.0449
GO:0030246	carbohydrate binding	12	40	0.0457
	**Cellular Component**			
GO:0042582	azurophil granule	4	4	0.0011
GO:0000790	nuclear chromatin	12	27	0.0014
GO:0031616	spindle pole centrosome	3	4	0.0209
GO:0000786	nucleosome	5	10	0.0223
GO:0042719	mitochondrial intermembrane space protein transporter complex	2	2	0.0333
GO:0001740	Barr body	2	2	0.0333
GO:0072687	meiotic spindle	2	2	0.0333
GO:0034751	aryl hydrocarbon receptor complex	2	2	0.0333
GO:0043196	varicosity	2	2	0.0333
GO:0001931	uropod	3	5	0.0453
**28°C+B3**				
	**Biological Process**			
GO:0003009	skeletal muscle contraction	5	7	0.0015
GO:0051897	positive regulation of protein kinase B signaling	6	11	0.0034
GO:0010677	negative regulation of cellular carbohydrate metabolic process	3	3	0.0039
GO:1901896	positive regulation of calcium-transporting ATPase activity	3	3	0.0039
GO:0032781	positive regulation of ATPase activity	9	15	0.0055
GO:0050873	brown fat cell differentiation	3	4	0.0138
GO:0006937	regulation of muscle contraction	8	22	0.0147
GO:0071560	cellular response to transforming growth factor beta stimulus	8	22	0.0147
GO:0060048	cardiac muscle contraction	6	15	0.0209
GO:0048193	Golgi vesicle transport	12	42	0.024
GO:1903630	regulation of aminoacyl-tRNA ligase activity	2	2	0.0249
GO:0070836	caveola assembly	2	2	0.0249
GO:1901017	negative regulation of potassium ion transmembrane transporter activity	2	2	0.0249
GO:0009226	nucleotide-sugar biosynthetic process	2	2	0.0249
GO:0071481	cellular response to X-ray	2	2	0.0249

GO:0031571	mitotic G1 DNA damage checkpoint	2	2	0.0249
GO:1904398	positive regulation of neuromuscular junction development	2	2	0.0249
GO:0035414	negative regulation of catenin import into nucleus	2	2	0.0249
GO:0030643	cellular phosphate ion homeostasis	2	2	0.0249
GO:0071378	cellular response to growth hormone stimulus	3	5	0.0305
GO:2000059	negative regulation of protein ubiquitination involved in ubiquitin-dependent protein catabolic process	3	5	0.0305
GO:0035774	positive regulation of insulin secretion involved in cellular response to glucose stimulus	3	5	0.0305
GO:0046627	negative regulation of insulin receptor signaling pathway	3	5	0.0305
GO:0032780	negative regulation of ATPase activity	3	5	0.0305
GO:0034260	negative regulation of GTPase activity	3	5	0.0305
GO:0043392	negative regulation of DNA binding	3	5	0.0305
GO:0008286	insulin receptor signaling pathway	7	14	0.0384
GO:0030518	intracellular steroid hormone receptor signaling pathway	6	17	0.0392
GO:0045913	positive regulation of carbohydrate metabolic process	4	9	0.0399
GO:0000086	G2/M transition of mitotic cell cycle	4	9	0.0399
GO:0097421	liver regeneration	5	13	0.0411
GO:0050772	positive regulation of axonogenesis	5	13	0.0411
GO:0007030	Golgi organization	7	22	0.046
	**Molecular Function**			
GO:0035259	glucocorticoid receptor binding	3	4	0.013
GO:0001671	ATPase activator activity	3	4	0.013
GO:0008134	transcription factor binding	15	55	0.016
GO:0050431	transforming growth factor beta binding	2	2	0.024
GO:0031730	CCR5 chemokine receptor binding	2	2	0.024
GO:0031014	troponin T binding	2	2	0.024
GO:0005044	scavenger receptor activity	3	5	0.029
GO:0019905	syntaxin binding	4	9	0.037
GO:0016779	nucleotidyltransferase activity	4	9	0.037
GO:0004888	transmembrane signaling receptor activity	4	9	0.037
	**Cellular Component**			
GO:0005887	integral component of plasma membrane	12	32	0.002
GO:0030134	COPII-coated ER to Golgi transport vesicle	5	9	0.0067
GO:0005861	troponin complex	2	2	0.0245
GO:0001741	XY body	2	2	0.0245
GO:0043596	nuclear replication fork	3	5	0.0299
GO:0044295	axonal growth cone	3	5	0.0299
GO:0016459	myosin complex	5	13	0.0399

Table S3. Full list of differentially regulated proteins in WAT.

GeneID	Gene Name	Logfc	Adjpv
**20°C**			
117028	Bin1	-2.79	8.97E-06

304290	Kdelr2	-2.68	4.47E-05
24667	Ppm1b	-2.65	0.000108
84114	Agps	-1.35	0.000125
84401	Puf60	-2.94	0.000753
300983	Abhd14b	0.94	0.000791
29218	Rcn2	-2.40	0.000854
290028	Osgep	-0.94	0.002064
24230	Tspo	-2.34	0.00248
171452	Rab3il1	-2.15	0.00629
84355	Atox1	-0.89	0.007345
311428	RGD1311739	1.07	0.007461
25246	Bsg	0.84	0.007876
25027	Slc16a1	1.02	0.008061
361999	Anp32e	-3.03	0.00862
24788	Sord	0.81	0.01132
301384	Hibch	1.64	0.013339
83576	Sort1	0.96	0.014054
29666	Psmb6	0.72	0.017559
690131	Hist2h2aa2	0.92	0.024289
29428	Celf2	-1.64	0.024896
84428	Dctn4	1.81	0.031509
54321	Cnn3	-1.91	0.032921
29528	Vamp3	0.68	0.034143
313200	Hsdl2	0.66	0.034361
25287	Acadl	0.67	0.03642
83472	Ugdh	0.61	0.041704
689284	Rpl38	-3.68	0.042184
24383	Gapdh	-0.72	0.046605
287633	Lrrc59	1.23	0.050259
25604	Pcmt1	-0.69	0.053192
681059	Vps25	-1.20	0.054081
79223	Gk	1.62	0.058056
691947	Eif3j	-0.97	0.062525
302500	Mcts1	1.98	0.065517
64517	Thop1	-1.02	0.066596
84472	Ilf3	-3.91	0.067026
65033	Stx12	0.65	0.06742
79131	Fabp3	2.67	0.067767
24172	Adh1	3.28	0.075818
114612	Ddx39b	-3.63	0.076071
369017	Krt5	2.80	0.076106
362809	Ptges3	-0.62	0.079947
54231	Car2	1.69	0.081896
171164	Gbp2	-0.65	0.08245
171155	Hadhb	0.65	0.087263
25104	Pc	0.63	0.087972
294673	Hexb	2.37	0.088946
65152	Pfkm	-2.15	0.091004
24918	Stat5a	1.42	0.094928
362115	Fam129b	0.99	0.106174
25725	Prkar1a	-1.02	0.106696

64362	Des	-0.74	0.108631
29651	Aldh1a7	3.17	0.111891
59108	Mb	-2.51	0.114158
25698	Ass1	3.41	0.115407
114123	Sardh	3.01	0.117946
65151	Rida	1.40	0.118124
84357	Sh3kbp1	-2.26	0.118967
24248	Cat	0.94	0.119545
295284	Rbm8a	-2.91	0.121692
83527	Dbnl	-2.21	0.122676
83805	Src	1.55	0.123643
363854	Elavl1	-0.67	0.125734
29271	Cfl1	-1.02	0.126378
307842	Vac14	-1.03	0.128208
294568	Wasf1	-2.01	0.128265
64526	Ech1	0.62	0.128887
113965	Hadh	0.89	0.131054
64679	Tgm4	-1.15	0.131104
683313	LOC683313	0.77	0.13306
63864	Hsd17b10	0.86	0.138671
299194	Ptgr2	1.16	0.138818
24307	Cyp4b1	-3.32	0.143961
297893	Hdac1	-1.83	0.151039
79248	Abca2	1.01	0.151314
300218	Tuba1c	-3.89	0.152971
81521	Msn	-0.84	0.160485
24666	Ppm1a	1.18	0.162655
102550391	LOC102550391	1.19	0.165852
64533	Pnpo	-4.31	0.166568
84478	Ufd1	0.87	0.172453
500419	Rmdn1	0.96	0.180088
24360	Fabp1	3.63	0.180799
100125372	Ces1f	-1.25	0.182806
24284	Csn1s1	2.46	0.186806
299923	Ndrg1	0.63	0.187013
24159	Acly	1.25	0.19101
681429	Rps27l	-0.63	0.193334
84509	Ran	-0.74	0.194937
246298	Retsat	0.97	0.195298
117130	Grifin	-2.17	0.195614
29474	Coro1b	-0.63	0.196282
500040	Tes	2.33	0.198406
116689	Ptpn6	-1.10	0.202346
25499	Nrdc	1.35	0.202642
678759	Ndufa10	-2.34	0.203597
683788	Fscn1	-0.70	0.20588
29459	Rbbp9	-1.22	0.207521
25106	Rgn	2.76	0.208098
64158	Tuba1a	5.02	0.218196
497811	Xdh	0.79	0.221165
25371	Adprh	-2.29	0.224889

306332	Ap1m1	0.71	0.227335
117282	Hnrnpk	-0.75	0.229928
296710	Arpc5l	-0.95	0.232611
29443	Ahcy	-0.90	0.23316
113940	Gmfg	-1.53	0.234475
24957	Glul	1.73	0.235712
60356	Csad	1.10	0.236915
24223	B2m	-0.64	0.238063
363425	Cav2	0.88	0.238385
50671	Fasn	1.48	0.239956
64040	Aldh9a1	0.62	0.241028
56781	Myl1	-3.64	0.241232
89827	Ddx39a	-0.78	0.244658
**28°C+B3**			
83730	Vamp8	-3.75	6.93E-05
29521	Scamp1	1.51	0.000111
25116	Hsd11b1	0.92	0.000382
117045	Eif4e	-0.69	0.000942
25342	Oxtr	1.79	0.001104
298566	C1qa	0.85	0.001124
445268	Ufc1	-0.65	0.001133
78947	Gcs1	0.61	0.00204
266734	Npas4	0.87	0.004234
246303	Serbp1	0.78	0.004516
25139	Slc2a4	1.36	0.005653
619574	LOC619574	-1.74	0.006446
64317	Gpx3	1.00	0.006558
84474	Ddx1	-0.73	0.007131
24471	Hspb1	1.26	0.007327
170673	Palm	2.47	0.007749
313035	Dnajc8	-2.09	0.008094
64045	Glrx	-3.30	0.008229
122799	Rps25	-1.04	0.009005
252928	Timm13	-0.62	0.009087
25611	Otc	0.72	0.009208
54319	Ezr	-2.37	0.009756
171114	Ndrg2	0.99	0.011103
64306	Rpl27	-0.99	0.013106
170520	Cygb	0.73	0.01364
85333	Slc25a4	0.74	0.014342
303606	Ccdc47	1.69	0.015281
81520	Marcksl1	-2.76	0.016098
116547	S100a8	2.76	0.018187
497009	Naaa	-3.00	0.018457
494345	Pdcd10	-0.80	0.020933
361663	Lhpp	-1.87	0.022049
25339	Npr3	1.07	0.022951
24233	C4a	0.96	0.023674
64152	Chp1	1.02	0.025969
300757	Hexa	-0.66	0.026564
361051	Phf11	-1.84	0.026791

260321	Fkbp4	-0.91	0.028603
292148	Eif3a	-0.71	0.030009
64028	Tsnax	-3.94	0.031875
100134871	LOC100134871	2.33	0.032522
117259	Tra2b	-1.82	0.032726
24648	Serpina1	0.98	0.033119
100911615	LOC100911615	1.62	0.03415
292925	Tsg101	-1.88	0.035767
300035	Pycr3	-0.66	0.036528
360882	Cadm3	1.85	0.036758
308650	Hnrnph2	-1.01	0.038168
58927	Rpl36	-0.84	0.039681
29635	Timm44	-2.58	0.041016
25282	Cox6a1	-1.73	0.041823
81504	Grb2	-1.13	0.043459
29360	Selenop	0.91	0.044436
29286	Rps17	-0.67	0.046061
24674	Ppp3ca	-1.18	0.04939
25524	Psap	-0.99	0.050226
64352	Gstm5	1.30	0.050372
116698	Trim28	-1.28	0.050847
24614	Orm1	1.57	0.051012
171133	Gcsh	3.37	0.051166
305679	Vcl	0.78	0.051276
25030	Andpro	2.99	0.051519
288001	Kng1	0.86	0.05194
64665	Flot1	0.79	0.053329
78958	Bcam	1.37	0.053481
681544	LOC681544	0.77	0.053624
24439	Hagh	1.13	0.056742
367562	Gaa	1.10	0.057099
58827	Mest	-0.74	0.057415
362401	Tmem43	0.61	0.060953
191574	Akr1c14	0.70	0.062102
294239	Ddah2	0.66	0.063782
24825	Tf	0.96	0.066528
65261	Myo1c	0.67	0.067612
58917	Hpx	0.65	0.068548
85332	Cavin3	0.87	0.069924
690050	Tpmt	-2.40	0.069953
60581	Acaca	-3.44	0.071
252929	Ctsz	-1.05	0.072146
363113	Syncrip	-0.63	0.072433
296709	Rpl35	-0.99	0.072466
300075	Tomm22	-0.76	0.07444
100359922	LOC100359922	-0.80	0.074441
29236	Rpsa	-0.68	0.074828
25420	Cryab	5.29	0.074881
83712	Rbbp7	-0.76	0.075646
25473	Lamb2	0.88	0.075929
29558	Fcgrt	0.80	0.076078

56780	Acpp	-1.89	0.078124
81775	Rps21	-1.16	0.078693
29669	Psma2	-0.61	0.078717
24440	Hbb	2.07	0.078843
360504	Hba2	0.96	0.079007
83783	Sult1a1	0.62	0.07972
117041	Nln	3.09	0.080074
25742	S100b	1.24	0.080926
300677	Atp5l	0.67	0.081587
246233	Macrod1	2.41	0.082923
64507	Fmod	2.19	0.083367
116549	Csnk2a1	-0.85	0.083542
499782	Rpl12	-0.67	0.085869
29283	Rpl29	-0.65	0.086577
108348260	LOC108348260	-0.73	0.092616
81766	Rpl18	-0.67	0.096475
29288	Rps3a	-0.79	0.097091
500538	Ybx1	-0.62	0.097152
360646	Limd2	-4.82	0.098828
360471	Usp7	-1.03	0.103854
364838	Reep5	1.15	0.105331
57341	Parva	0.76	0.106336
171137	Khsrp	-1.01	0.10724
315218	Lmf2	0.95	0.108704
25035	Cyb5r3	1.02	0.110565
116662	Ecm1	1.41	0.111825
171577	Epcam	-1.15	0.112584
307779	Rbmxrtl	-0.96	0.115081
307947	Set	-2.08	0.117698
60571	Mybbp1a	-1.40	0.11861
64031	Pdcd4	-1.13	0.119102
65984	Aacs	-3.23	0.120908
362855	Rtcb	-0.61	0.121081
56611	Anxa2	0.97	0.122294
296596	Rpl7a	-0.70	0.12345
114113	Pafah1b3	-1.02	0.12414
369016	Myadm	0.73	0.124691
28298	Rpl32	-1.06	0.125753
450225	Krt10	1.17	0.127176
59114	Slc9a3r1	-1.09	0.12891
25757	Cpt1a	3.07	0.12931
317381	Ccdc22	5.59	0.129886
85255	Hacl1	-2.44	0.130084
302562	Plp2	0.71	0.131025
362631	Rpl11	-0.75	0.132254
691531	Rps28	-0.87	0.133683
79224	Serpind1	0.66	0.134675
192276	Coro7	-1.16	0.136285
81681	Lss	1.38	0.141036
55939	Apom	0.63	0.141553
81729	Rpl10a	-0.98	0.142368

360626	Krt19	-2.95	0.142817
291434	Rpl17	-0.66	0.143666
305343	Pds5a	-3.37	0.145504
25419	Crp	0.87	0.147213
290651	Isyna1	-1.36	0.148286
360854	Arpc5	-0.76	0.151956
80846	Hnrnpl	-1.05	0.153574
300079	Rpl3	-0.81	0.153618
50664	Gnao1	0.91	0.153753
29491	Itsn1	-3.57	0.153827
113936	Cpb2	0.93	0.154271
100360522	LOC100360522	-1.22	0.155548
79256	Hnrnpd	-1.69	0.15639
360576	Tusc5	1.02	0.158706
308384	Sae1	-1.99	0.159931
296654	Gsn	0.87	0.160988
361673	Ifitm3	-1.59	0.165339
29389	Tnni2	-4.01	0.165619
29671	Psma4	-0.73	0.166824
65204	Cnn1	2.94	0.168593
301252	Hsp90ab1	-0.75	0.170109
24786	Sod1	-0.92	0.170236
81008	Itga7	1.13	0.170372
117557	Tpm3	-0.84	0.173177
497794	Mug1	0.61	0.174553
58952	Cpq	0.62	0.175249
114499	Hdgf	-0.82	0.178124
65137	Ruvbl1	-1.53	0.178135
25010	Scgb2a1	4.76	0.179267
117042	Rpl6	-0.79	0.180849
287191	Rars	-0.82	0.181074
25686	Gnai1	0.63	0.183971
294853	Krt18	-2.59	0.185246
286938	Gimap4	-1.75	0.186195
29648	Nudc	-0.86	0.186791
85496	Enpp1	-1.97	0.18985
83502	Cdh1	-1.92	0.19445
25330	Lipe	1.13	0.195171
83510	Lypla2	-0.91	0.195635
29473	Aoc3	0.70	0.195941
117280	Hnrnpu	-1.07	0.196818
116655	Hnrnpm	-1.12	0.200116
25126	Stat5b	-0.87	0.200585
24366	Fgb	0.86	0.200904
108350501	LOC108350501	-1.35	0.202062
64205	Rplp0	-0.79	0.205181
79116	Apex1	-3.25	0.206758
25368	Adk	-0.72	0.210856
290641	Rpl18a	-0.77	0.211728
170724	Anp32b	-1.02	0.212806
297699	Strap	-0.70	0.213497

100362830	LOC100362830	-0.77	0.218146
24968	Psmb8	-0.84	0.219647
252922	Pzp	0.96	0.223427
291983	Psmb10	-1.07	0.22378
116685	Lmnb1	-1.10	0.224812
361512	Ehd2	1.19	0.227089
25269	Pvalb	2.17	0.227186
108348062	LOC108348062	-2.51	0.228587
29563	Crabp2	-3.47	0.238336
64347	Sncg	1.49	0.23862
25292	Apoc1	2.25	0.239531
309187	Atl3	0.84	0.240149
290644	Ifi30	-0.70	0.240249
24346	Ces1c	0.91	0.243417
103690821	LOC103690821	-0.66	0.245662
293692	Ehd1	1.02	0.247676

Table S4. Full list of GO terms enriched in WAT

Goid	Goname	Countde	Countall	Pv_Elim
**20°C**				
**Biological Process**				
GO:0030330	DNA damage response, signal transduction by p53 class mediator	5	6	0.0022
GO:0048711	positive regulation of astrocyte differentiation	3	3	0.0023
GO:0071498	cellular response to fluid shear stress	3	3	0.0023
GO:0032780	negative regulation of ATPase activity	3	3	0.0023
GO:0051607	defense response to virus	5	9	0.003
GO:0001822	kidney development	11	35	0.0036
GO:0050731	positive regulation of peptidyl-tyrosine phosphorylation	7	17	0.0037
GO:0002244	hematopoietic progenitor cell differentiation	3	4	0.0081
GO:0045577	regulation of B cell differentiation	3	4	0.0081
GO:0042130	negative regulation of T cell proliferation	3	4	0.0081
GO:0000077	DNA damage checkpoint	3	4	0.0081
GO:0002763	positive regulation of myeloid leukocyte differentiation	3	4	0.0081
GO:0071803	positive regulation of podosome assembly	3	4	0.0081
GO:0016477	cell migration	26	127	0.0104

GO:0034314	Arp2/3 complex-mediated actin nucleation	8	13	0.0124
GO:0010592	positive regulation of lamellipodium assembly	4	8	0.0133
GO:0120033	negative regulation of plasma membrane bounded cell projection assembly	4	8	0.0133
GO:0051289	protein homotetramerization	8	26	0.0147
GO:1902743	regulation of lamellipodium organization	8	13	0.0164
GO:2000279	negative regulation of DNA biosynthetic process	4	5	0.017
GO:0000245	spliceosomal complex assembly	4	5	0.017
GO:2000573	positive regulation of DNA biosynthetic process	6	17	0.017
GO:1902570	protein localization to nucleolus	2	2	0.0173
GO:0032480	negative regulation of type I interferon production	2	2	0.0173
GO:0071362	cellular response to ether	2	2	0.0173
GO:0034616	response to laminar fluid shear stress	2	2	0.0173
GO:0006499	N-terminal protein myristoylation	2	2	0.0173
GO:0097484	dendrite extension	2	2	0.0173
GO:0035855	megakaryocyte development	2	2	0.0173
GO:0099601	regulation of neurotransmitter receptor activity	2	2	0.0173
GO:0035970	peptidyl-threonine dephosphorylation	2	2	0.0173
GO:2000394	positive regulation of lamellipodium morphogenesis	2	2	0.0173
GO:0010870	positive regulation of receptor biosynthetic process	2	2	0.0173
GO:0031954	positive regulation of protein autophosphorylation	2	2	0.0173
GO:0060740	prostate gland epithelium morphogenesis	2	2	0.0173
GO:2000601	positive regulation of Arp2/3 complex-mediated actin nucleation	2	2	0.0173
GO:1902463	protein localization to cell leading edge	2	2	0.0173

GO:2000107	negative regulation of leukocyte apoptotic process	3	5	0.0184
GO:0048011	neurotrophin TRK receptor signaling pathway	3	5	0.0184
GO:0042475	odontogenesis of dentin-containing tooth	3	5	0.0184
GO:0051701	interaction with host	8	27	0.0187
GO:0030203	glycosaminoglycan metabolic process	4	9	0.0215
GO:0050792	regulation of viral process	8	28	0.0232
GO:0051091	positive regulation of DNA binding transcription factor activity	7	23	0.0236
**Molecular Function**				
GO:0051287	NAD binding	11	31	0.0013
GO:0008144	drug binding	9	29	0.0101
GO:0005001	transmembrane receptor protein tyrosine phosphatase activity	2	2	0.0178
GO:0005521	lamin binding	3	5	0.0191
GO:0042393	histone binding	5	13	0.021
GO:0033613	activating transcription factor binding	3	6	0.0345
GO:0003857	3-hydroxyacyl-CoA dehydrogenase activity	3	6	0.0345
GO:0045296	cadherin binding	22	113	0.0365
GO:0004028	3-chloroallyl aldehyde dehydrogenase activity	2	3	0.0486
GO:0071933	Arp2/3 complex binding	2	3	0.0486
GO:0004854	xanthine dehydrogenase activity	2	3	0.0486
GO:0003785	actin monomer binding	2	3	0.0486
GO:0046912	transferase activity, transferring acyl groups, acyl groups converted into alkyl on transfer	2	3	0.0486
GO:0005324	long-chain fatty acid transporter activity	2	3	0.0486
GO:0005540	hyaluronic acid binding	2	3	0.0486
**Cellular Component**				
GO:0005884	actin filament	9	24	0.0021
GO:0032993	protein-DNA complex	5	11	0.0088
GO:0002102	podosome	7	14	0.013
GO:0016607	nuclear speck	8	26	0.0146
GO:0031209	SCAR complex	2	2	0.0172

GO:0042611	MHC protein complex	2	2	0.0172
GO:0005687	U4 snRNP	2	2	0.0172
GO:0005856	cytoskeleton	49	238	0.0284
GO:0030054	cell junction	41	216	0.0291
GO:0005681	spliceosomal complex	9	22	0.0311
GO:0031258	lamellipodium membrane	3	6	0.033
GO:0071437	invadopodium	3	6	0.033
GO:0005912	adherens junction	29	162	0.0408
GO:0000407	pre-autophagosomal structure	2	3	0.0471
GO:0002080	acrosomal membrane	2	3	0.0471
**28°C+B3**				
**Biological Process**				
GO:0006953	acute-phase response	7	12	0.0035
GO:0000381	regulation of alternative mRNA splicing, via spliceosome	7	12	0.0035
GO:0034113	heterotypic cell-cell adhesion	8	12	0.0061
GO:0070528	protein kinase C signaling	4	5	0.0063
GO:0015671	oxygen transport	4	5	0.0063
GO:1901741	positive regulation of myoblast fusion	3	3	0.0077
GO:0070934	CRD-mediated mRNA stabilization	3	3	0.0077
GO:0007566	embryo implantation	6	12	0.0179
GO:0040007	growth	29	103	0.0209
GO:0071345	cellular response to cytokine stimulus	25	86	0.0211
GO:0009059	macromolecule biosynthetic process	84	332	0.0239
GO:0070293	renal absorption	3	4	0.0261
GO:0071392	cellular response to estradiol stimulus	3	4	0.0261
GO:0042993	positive regulation of transcription factor import into nucleus	3	4	0.0261
GO:0048821	erythrocyte development	3	4	0.0261
GO:0046597	negative regulation of viral entry into host cell	3	4	0.0261
GO:0044319	wound healing, spreading of cells	3	4	0.0261
GO:0043516	regulation of DNA damage response, signal transduction by p53 class mediator	3	4	0.0261
GO:0031953	negative regulation of protein autophosphorylation	3	4	0.0261

GO:2000648	positive regulation of stem cell proliferation	3	4	0.0261
GO:1900087	positive regulation of G1/S transition of mitotic cell cycle	3	4	0.0261
GO:0042273	ribosomal large subunit biogenesis	8	15	0.027
GO:0030032	lamellipodium assembly	6	13	0.0278
GO:0006281	DNA repair	10	22	0.0281
GO:0032103	positive regulation of response to external stimulus	11	31	0.0287
GO:0051241	negative regulation of multicellular organismal process	33	123	0.0288
GO:0042255	ribosome assembly	8	20	0.0291
GO:0042307	positive regulation of protein import into nucleus	7	11	0.0307
GO:0070670	response to interleukin-4	5	10	0.0308
GO:0072659	protein localization to plasma membrane	15	47	0.0309
GO:0071407	cellular response to organic cyclic compound	33	103	0.0311
GO:0034114	regulation of heterotypic cell-cell adhesion	4	7	0.0317
GO:0042755	eating behavior	4	7	0.0317
GO:2001235	positive regulation of apoptotic signaling pathway	9	24	0.0325
GO:0019915	lipid storage	7	17	0.0346
GO:0051090	regulation of DNA binding transcription factor activity	10	28	0.0348
GO:0090316	positive regulation of intracellular protein transport	15	32	0.0354
GO:0045861	negative regulation of proteolysis	21	67	0.0377
GO:0060088	auditory receptor cell stereocilium organization	2	2	0.039
GO:0032415	regulation of sodium:proton antiporter activity	2	2	0.039
GO:0061158	3'-UTR-mediated mRNA destabilization	2	2	0.039
GO:2001014	regulation of skeletal muscle cell differentiation	2	2	0.039
GO:0050884	neuromuscular process controlling posture	2	2	0.039
GO:2001026	regulation of endothelial cell chemotaxis	2	2	0.039

GO:0019731	antibacterial humoral response	2	2	0.039
GO:0071386	cellular response to corticosterone stimulus	2	2	0.039
GO:0006388	tRNA splicing, via endonucleolytic cleavage and ligation	2	2	0.039
GO:2000047	regulation of cell-cell adhesion mediated by cadherin	2	2	0.039
GO:0006407	rRNA export from nucleus	2	2	0.039
GO:2001137	positive regulation of endocytic recycling	2	2	0.039
GO:0019886	antigen processing and presentation of exogenous peptide antigen via MHC class II	2	2	0.039
GO:0060355	positive regulation of cell adhesion molecule production	2	2	0.039
GO:0002523	leukocyte migration involved in inflammatory response	2	2	0.039
GO:0031643	positive regulation of myelination	2	2	0.039
GO:0072697	protein localization to cell cortex	2	2	0.039
GO:0010642	negative regulation of platelet-derived growth factor receptor signaling pathway	2	2	0.039
GO:0080111	DNA demethylation	2	2	0.039
GO:0052405	negative regulation by host of symbiont molecular function	2	2	0.039
GO:1905063	regulation of vascular smooth muscle cell differentiation	2	2	0.039
GO:0030643	cellular phosphate ion homeostasis	2	2	0.039
GO:0010757	negative regulation of plasminogen activation	2	2	0.039
GO:0071763	nuclear membrane organization	2	2	0.039
GO:0060669	embryonic placenta morphogenesis	2	2	0.039
GO:0035176	social behavior	2	2	0.039
GO:0018065	protein-cofactor linkage	2	2	0.039
GO:0015886	heme transport	2	2	0.039
GO:0050427	3'-phosphoadenosine 5'-phosphosulfate metabolic process	2	2	0.039

GO:0002925	positive regulation of humoral immune response mediated by circulating immunoglobulin	2	2	0.039
GO:0010998	regulation of translational initiation by eIF2 alpha phosphorylation	2	2	0.039
GO:0007156	homophilic cell adhesion via plasma membrane adhesion molecules	2	2	0.039
GO:0000387	spliceosomal snRNP assembly	2	2	0.039
GO:0007194	negative regulation of adenylate cyclase activity	2	2	0.039
GO:1904401	cellular response to Thyroid stimulating hormone	2	2	0.039
GO:0005984	disaccharide metabolic process	2	2	0.039
GO:0000447	endonucleolytic cleavage in ITS1 to separate SSU-rRNA from 5.8S rRNA and LSU-rRNA from tricistronic rRNA transcript (SSU-rRNA, 5.8S rRNA, LSU-rRNA)	2	2	0.039
GO:0000461	endonucleolytic cleavage to generate mature 3'-end of SSU-rRNA from (SSU-rRNA, 5.8S rRNA, LSU-rRNA)	2	2	0.039
GO:0000463	maturation of LSU-rRNA from tricistronic rRNA transcript (SSU-rRNA, 5.8S rRNA, LSU-rRNA)	2	2	0.039
GO:0038089	positive regulation of cell migration by vascular endothelial growth factor signaling pathway	2	2	0.039
GO:1903533	regulation of protein targeting	6	14	0.0408
GO:0000165	MAPK cascade	22	78	0.0414
GO:0022409	positive regulation of cell-cell adhesion	9	25	0.0423
GO:0006396	RNA processing	31	75	0.0432
GO:0045596	negative regulation of cell differentiation	19	66	0.0463
GO:0032092	positive regulation of protein binding	7	18	0.0474
GO:0051053	negative regulation of DNA metabolic process	5	11	0.0475

GO:0007188	adenylate cyclase-modulating G-protein coupled receptor signaling pathway	5	11	0.0475
GO:0034381	plasma lipoprotein particle clearance	5	11	0.0475
GO:0008154	actin polymerization or depolymerization	17	42	0.0483
**Molecular Function**				
GO:0003735	structural constituent of ribosome	24	62	0.00037
GO:0005344	oxygen carrier activity	4	5	0.00657
GO:0003730	mRNA 3'-UTR binding	8	18	0.01548
GO:0003682	chromatin binding	10	25	0.01623
GO:0140097	catalytic activity, acting on DNA	5	9	0.01908
GO:0042162	telomeric DNA binding	3	4	0.02688
GO:0045294	alpha-catenin binding	3	4	0.02688
GO:0004527	exonuclease activity	3	4	0.02688
GO:0003723	RNA binding	73	281	0.02734
GO:0019825	oxygen binding	4	7	0.03278
GO:0031720	haptoglobin binding	2	2	0.03976
GO:0001091	RNA polymerase II basal transcription factor binding	2	2	0.03976
GO:0001105	RNA polymerase II transcription coactivator activity	2	2	0.03976
GO:0015926	glucosidase activity	2	2	0.03976
GO:0005055	laminin receptor activity	2	2	0.03976
GO:0005089	Rho guanyl-nucleotide exchange factor activity	2	2	0.03976
GO:0045159	myosin II binding	2	2	0.03976
GO:0043395	heparan sulfate proteoglycan binding	2	2	0.03976
GO:0046790	virion binding	2	2	0.03976
GO:0004888	transmembrane signaling receptor activity	7	13	0.04822
GO:0070851	growth factor receptor binding	5	11	0.04938
**Cellular Component**				
GO:0022625	cytosolic large ribosomal subunit	15	30	0.00018
GO:0016323	basolateral plasma membrane	14	32	0.00164
GO:0005833	hemoglobin complex	3	3	0.00782
GO:0005903	brush border	10	23	0.00812
GO:0030864	cortical actin cytoskeleton	9	20	0.00918
GO:0016327	apicolateral plasma membrane	3	4	0.02666
GO:0044451	nucleoplasm part	18	58	0.0269

GO:0005637	nuclear inner membrane	5	10	0.03167
GO:0035770	ribonucleoprotein granule	11	32	0.03804
GO:0097225	sperm midpiece	2	2	0.03953
GO:0032426	stereocilium tip	2	2	0.03953
GO:0061827	sperm head	2	2	0.03953
GO:0070937	CRD-mediated mRNA stability complex	2	2	0.03953
GO:0072669	tRNA-splicing ligase complex	2	2	0.03953
GO:0071204	histone pre-mRNA 3'end processing complex	2	2	0.03953
GO:0034451	centriolar satellite	2	2	0.03953
GO:0030686	90S preribosome	2	2	0.03953
